# Spontaneous Heterotopic Pregnancy Associated with Massive Intraperitoneal Haemorrhage and a Normal Heart Rate, Illustrating the Concept of Relative Bradycardia

**DOI:** 10.1155/2019/2893149

**Published:** 2019-03-18

**Authors:** Charles Gallaher, Farshad Tahmasebi, Ahmad Sayasneh, Gautam Mehra

**Affiliations:** ^1^Emergency Department, St Helier Hospital, Wrythe Ln, Sutton, Carshalton SM5 1AA, UK; ^2^Obstetrics and Gynaecology, Guy's & St Thomas' NHS Foundation Trust, Westminster Bridge Rd, London SE1 7EH, UK

## Abstract

A 28-year-old, 9 and a half weeks pregnant (spontaneous conception) multigravida presented with abdominal pain and vaginal bleeding. On examination, her abdomen was diffusely tender, particularly in the right iliac fossa, though guarding was absent. Transabdominal and transvaginal ultrasonography demonstrated a viable intrauterine pregnancy and large-volume intraperitoneal haemoperitoneum; the right ovary could not be identified. The patient became hypotensive with decreased responsiveness, yet her heart rate remained normal. She proceeded to surgery where a ruptured right tubal ectopic pregnancy was identified and right salpingectomy was performed. Estimated blood loss was 3900ml. Postoperative recovery was uneventful. Ultrasound 3 days after surgery demonstrated a viable intrauterine pregnancy of gestational age 9 weeks + 1 day. The patient remains well. Her anomaly scan at 20 weeks and 6 days showed normal growth, amniotic fluid, and Dopplers with no obvious structural defects. She is currently 27 weeks pregnant and will be rescanned at 36 weeks.

## 1. Introduction

Across the world, health professionals are trained to estimate severity of haemorrhage in accordance with the clinical criteria set out in the American College of Surgeons Advanced Trauma Life Support® (ATLS®) classification of haemorrhage [1]. This classifies haemorrhage into 4 classes (classes I-IV) based on clinical signs and is described in the text as “a useful tool for estimating the percentage of acute blood loss.” Class I haemorrhage is defined as up to 15% blood volume loss; class II haemorrhage = 15-30% blood volume loss; class III haemorrhage = 30-40% blood volume loss; and class IV haemorrhage = more than 40% blood volume loss.

According to the ATLS® classification of haemorrhage, class IV haemorrhage – which for a 70kg patient refers to blood volume loss of >2000ml – is associated with the following:

- heart rate > 140 beats per minute (bpm)
- decreased systolic blood pressure
- decreased pulse pressure
- respiratory rate > 35 breaths per minute
- negligible urine output
- confusion and lethargy

We present a case where, with one exception (heart rate was 112 bpm on first arrival in the emergency department), a patient with severe intraperitoneal haemorrhage remained persistently normocardic, with a heart rate of around 70 bpm, until after anaesthesia had been induced.

The MBRRACE-UK confidential enquiry into maternal deaths (2016) highlights the significance of early diagnosis of ectopic pregnancy to reduce maternal mortality [2]. The diagnosis and treatment of ectopic pregnancy can be challenging, especially when this presents atypically following tubal rupture in a spontaneous heterotopic pregnancy which in itself is rare.

## 2. Case Presentation

A 28-year-old multigravida (gravida 2, para 1), weighing approximately 90kg, presented at 9 and a half weeks with an 18-hour history of severe right iliac fossa pain, associated with brownish vaginal discharge, dysuria, diarrhoea, light-headedness, and feeling shivery. She was afebrile and did not complain of shoulder tip pain.

Past obstetric and gynaecological history included one full-term caesarean section; there was no history of tubal or other gynaecological surgery, sexually transmitted disease, endometriosis, or subfertility treatment, nor was there a history of intrauterine contraceptive device or progestogen-only contraceptive use.

Past medical history was otherwise unremarkable. The patient was on no regular medications and was an ex-smoker, and social history was otherwise unremarkable.

On examination, the patient looked to be in pain. Her abdomen was diffusely tender, particularly in the right iliac fossa. There was no loin tenderness, no abdominal guarding, and no rebound tenderness. Pelvic examination revealed right adnexal tenderness and cervical excitation; on speculum examination, the cervix was closed and brownish discharge was noted.

Vital signs from first assessment by the ambulance service onwards are displayed in Table 1.

### 2.1. Investigations

Urine dip was positive for protein (+), blood (++++), ketones (+++), leucocyte esterase (+), and qualitative beta-human chorionic gonadotrophin (beta-hCG).

Venous blood gas on arrival in the emergency department (ED) showed pH 7.419 (7.350-7.450), partial pressure of carbon dioxide (pCO2) 3.97 kPa, base excess (BE) -4.5 mmol/L, corrected bicarbonate (cHCO3^−^) 20.7 mmol/L, lactate 1.8 mmol/L (0.4-2.2 mmol/L), glucose 6.9 mmol/L (3.3-6.1 mmol/L), haemoglobin (Hb) 128 g/L (120-150 g/L), and haematocrit (Hct) 0.397 (0.360-0.470).

Laboratory blood tests on arrival in ED were as follows: total white cell count 14.1 x10^9^/L (4.0-11.0 x10^9^/L), neutrophils 11.1 x10^9^/L (1.5-7.0 x10^9^/L), Hb 130 g/L, Hct 0.375, sodium 138 mmol/L (135-145 mmol/L), potassium 4.0 mmol/L (3.5-5.0 mmol/L), creatinine 48 *μ*mol/L (45-84 *μ*mol/L), albumin 39 g/L (40-52 g/L), C-reactive protein 9 mg/L (0-4 mg/L), serum beta-hCG 133,561 IU/L, and HIV antibody screen negative.

On bedside transabdominal gynaecological ultrasound scan, performed in the ED approximately 2 hours after arrival, a 3.5cm intrauterine gestational sac was noted.

Formal gynaecological transabdominal ultrasound scan and Focused Assessment for Free Fluid (FAFF) ultrasound scan were performed on the early pregnancy unit approximately 3.5 hours after arrival in the ED, and showed a moderate amount of free fluid in the upper abdomen. Heterogeneous, avascular material was noted in the adnexa, in keeping with haemoperitoneum. The right ovary could not be identified, the left ovary appeared normal. An intrauterine gestational sac was noted.

 Transvaginal ultrasound scan was performed immediately afterwards and showed a viable intrauterine pregnancy, with gestational sac 140mm, and crown-rump length 9mm. The adnexa was difficult to assess.

 Arterial blood gas on high flow oxygen via nonrebreathe mask was performed when the patient became acutely unwell immediately following the above scan, and showed pH 7.403, pCO2 3.78 (4.67-6.00), pO2 35.86, BE -6.4, cHCO3^−^ 19.2, lactate 1.5, glucose 6.4, Hb 102 g/L, and Hct 0.315 (0.350-0.500).

### 2.2. Differential Diagnosis

Haemoperitoneum was diagnosed, with a differential diagnosis of either a ruptured spontaneous heterotopic pregnancy or a bleeding haemorrhagic corpus luteal cyst.

### 2.3. Treatment

In the scanning room on the early pregnancy unit, 4 hours after arrival in the ED, the patient complained of worsening, predominantly right-sided pain, and dizziness. She was re-examined and found to be cold and clammy with increased abdominal distension but no guarding. Vital signs recorded at the time were oxygen saturations of 100% on 2 litres of oxygen via nasal cannulae, heart rate of 76 bpm, and blood pressure of 90/59 mmHg. The patient became less responsive and the hospital medical emergency team was summoned. Intravenous crystalloid fluids had deliberately been withheld until this point in keeping with a hypotensive resuscitation strategy.

The patient was taken to the operating theatre where anaesthesia was induced approximately 6 hours after the patient's arrival in the ED. Having surgeons with advanced laparoscopic skills available, the decision was made to proceed with laparoscopic surgery (in most cases, surgeons might proceed with laparotomy).

**Figure 1:**
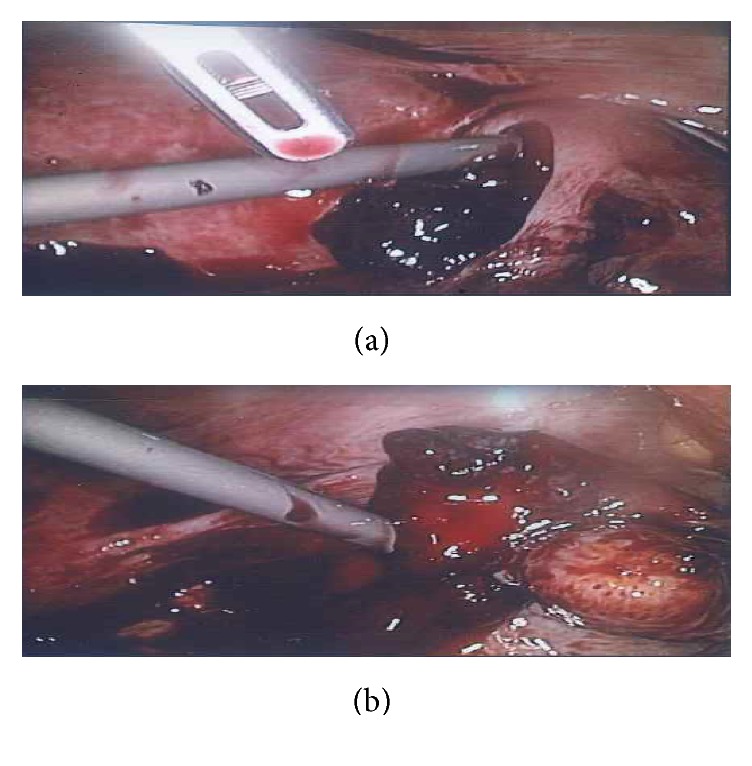
Ruptured right ectopic pregnancy.

Laparoscopic right salpingectomy and peritoneal lavage were performed (Figures 1(a) and 1(b)). Two units of red blood cells were transfused.

Summary of operative findings and procedures is as follows:

- Left ovary/tube normal
- Right tube engorged with rupture at isthmo-ampullary junction with extruding trophoblastic tissue and active bleeding from site of rupture
- 3900ml haemoperitoneum with large clots in pelvis, paracolic gutters, and upper abdomen
- Right tubal ectopic located and right salpingectomy performed
- Robinson's drain inserted to allow remaining blood to drain

### 2.4. Outcome and Follow-Up

Postoperative recovery was uneventful. Hb and haematocrit reached a nadir of 76 g/L and 0.226 2 days after surgery (Hb and haematocrit were 130g/L and 0.375 on arrival in the ED, and 2 units of red cells were transfused intraoperatively); this time lag is in keeping with the known delayed fall in Hb after haemorrhage.

**Figure 2:**
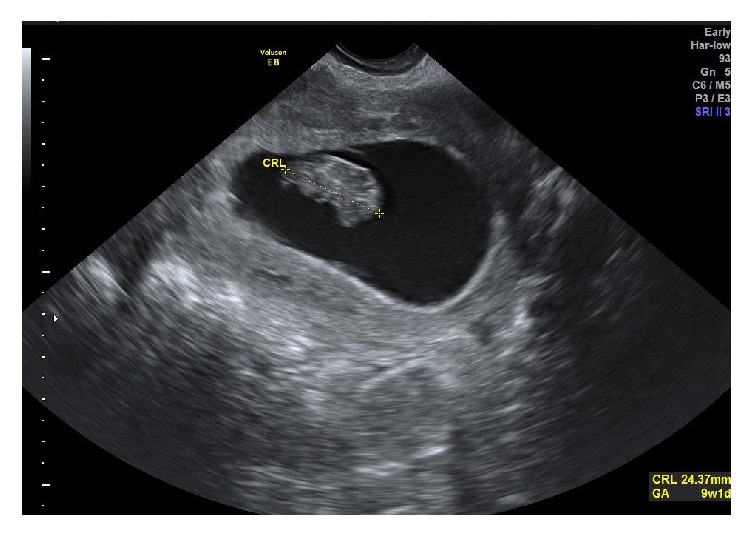
Intrauterine pregnancy demonstrated by transvaginal scan.

Formal early pregnancy ultrasound scan performed 3 days post-operatively showed a viable intrauterine pregnancy of gestational age 9 weeks + 1 day (Figure 2).

The patient was discharged from hospital 3 days after surgery, with a plan for a follow-up ultrasound scan in 2 weeks' time and iron supplementation.

Despite being critically ill with massive blood loss, her intrauterine pregnancy has developed normally. She is currently 27 weeks pregnant. Her routine dating and anomaly scans (performed at 12 weeks + 6 days and 20 + 6 days, respectively) demonstrated normal growth, amniotic fluid index, and uterine artery Dopplers.

She is to have early ultrasonography in future pregnancies.

## 3. Discussion

Heterotopic pregnancy—dizygotic twins, where one implants in the endometrial lining of the uterus and one outside of this location (i.e., ectopically)—is rare in spontaneous pregnancies (figures of approximately 1 in 4000 to 1 in 30,000 pregnancies are commonly quoted) [3–6]; it is far commoner if assisted reproductive technologies are used, with an incidence of approximately 1% [7, 8].

Barring pregnancies where assisted reproductive technologies have been used (where risk is disproportionately high), risk factors for heterotopic pregnancy are the same as for ectopic pregnancy [9, 10]. Our patient had no known risk factors except for being an ex-smoker.

What is particularly noteworthy about this case is the failure of the patient to mount a sustained tachycardia, despite massive intraperitoneal blood loss of nearly 4 litres.

This physiological response to haemorrhage runs counter to the above-described ATLS® principles, which are widely taught and internalised by health professionals across the world. A short-cut review was performed using 8 separate PubMed keyword searches, as follows:

1. “heterotopic pregnancy tachycardia”
  - 3 search results, 1 relevant paper
2. “heterotopic pregnancy bradycardia”
  - 1 search result, 1 relevant paper
3. “ectopic pregnancy tachycardia”
  - 95 search results, 7 relevant papers
4. “ectopic pregnancy bradycardia”
  - 27 search results, 3 relevant papers
5. “relative bradycardia haemorrhage” (includes results for “relative bradycardia hemorrhage”)
  - 43 search results, 3 relevant papers
6. “relative bradycardia abdom*∗*”
  - 43 search results, 1 relevant paper
7. “relative bradycardia shock”
  - 47 search results, 1 relevant paper
8. “paradoxical bradycardia”
  - 158 search results, 6 relevant papers

All bar 3 relevant papers had abstracts available, and all these abstracts were in English. One French-language paper with an English abstract was analysed in detail. The papers comprised single case reports, (sometimes large) case series, retrospective chart reviews, and retrospective analyses of trauma registries. No relevant experimental studies were found.

Precise definitions of “relative bradycardia” (also known as “paradoxical bradycardia”) vary. Quantitative definitions include

- HR <90 bpm with systolic BP < 90mmHg [11] and
- HR <100 bpm with systolic BP < 100mmHg [12].

Qualitative definitions include “the inability of the heart to respond to shock with tachycardia”[11] and “hypotension and the lack of a tachycardic response”[13].

Jansen described relative bradycardia as a sign of acute intraperitoneal bleeding in 1978 [14]. He presented 4 cases of women with intraperitoneal bleeding of pelvic origin who demonstrated relative bradycardia, becoming symptomatically hypotensive with a pulse rate of <80 bpm. It is posited that the observed relative bradycardia is due to reflex, parasympathetically mediated vasomotor disturbance in response to irritation of the peritoneum by recently shed blood (hence the frequently observed syncope and presyncope in cases of acute intraperitoneal haemorrhage of wide-ranging aetiologies). Jansen concluded that the phenomenon of relative bradycardia in acute intraperitoneal haemorrhage potentially causes diagnostic difficulty and disproportionately severe hypotension.

Snyder, in a retrospective chart review of 154 patients with documented ectopic pregnancy, found that, of 20 patients with systolic BP ≤90mmHg, 11 (55%) had a HR <100 bpm [15]. The quantity of haemoperitoneum did not correlate with the observed haemodynamic response. Again, a parasympathetic reflex response was thought to explain the inappropriately slow heart rate for the degree of hypotension. This theory is supported by Oberg and Thoren, who correlated increased activity in vagal cardiac afferent nerves with the appearance of reflex bradycardia during severe haemorrhage [16].

Mathlouthi et al. examined the correlation between vital signs and haemoperitoneum in ruptured ectopic pregnancy on a retrospective sample of 32 patients [17]. Mean maximum HR was 81.5 bpm (range: 70-140 bpm), with a mean systolic BP of 109mmHg (range: 70-150mmHg) for a mean haemoperitoneum of 694ml (range: 100-2000ml). Correlation between vital signs and volume of haemoperitoneum was poor. Hypotension was associated with blood loss of at least 1280ml. In only two cases were hypotension and tachycardia seen together, and correlation between tachycardia and hypotension was poor.

Somers, Spears, Maynard, and Syverud present a case of heterotopic pregnancy with relative bradycardia [18]. Cobb describes a similar case of ectopic pregnancy [19].

Several papers describe a similar relative bradycardia concept in the context of trauma [11, 20–22].

Clearly, failure to recognise the association of intraperitoneal haemorrhage with relative bradycardia may have patient safety implications. Fortunately, our patient recovered well, but the sheer volume of haemoperitoneum found at laparoscopy raises concerns that had her surgery been performed later, her outcome may not have been positive.

Other authors to have commented on this potential patient safety issue include Sucov, Deveau, Feola, and Sculli, who present a case of heterotopic pregnancy where diagnosis was delayed, “potentially because of lack of tachycardia associated with the hypotension”[9]. Adams and Greene report 5 cases of intraperitoneal haemorrhage in young healthy women associated with hypotension and lack of tachycardia [13]. They comment that this may delay definitive treatment by confusing the clinical picture.

Hick, Rodgerson, Heegaard, and Sterner, in a retrospective chart review of 51 cases of ectopic pregnancy, found that patients with normal vital signs had a 20% chance of having class IV blood loss at surgery, lending further support to the above patient safety concerns [23].

At first glance, it is perhaps hard to credit that our patient suffered such massive blood loss, given that her Hb on arrival was 130g/L and reached a nadir of 76g/L. However, the observed figure of 3900ml for measured blood loss is similar to predicted blood loss based on previous data. Lata et al. measured the fall in Hb/haematocrit associated with bleeding in obstetric patients [24]. Based on their data, a fall in Hb of 1g/L in obstetric patients results from every 47ml of blood loss. Our patient had a fall in Hb of 54g/L after being transfused 2 units. Given that 1 unit of red cells should increase Hb by 6g/L in a 90 kg female, the true fall in Hb is estimated at 66g/L [25]. This corresponds to a theoretical blood loss of 3100ml. However, our patient's large body habitus means that she is likely to have exhibited a smaller drop in Hb for a given volume of blood loss, raising the theoretical blood loss value for a given Hb drop further. The authors therefore believe that the observed 3900ml actual blood loss figure is likely to have been reasonably accurate.

Finally, it should not be forgotten that, in heterotopic pregnancy, maternal hypotension occurring during acute haemorrhage from rupture of the ectopic pregnancy may compromise perfusion to the intrauterine pregnancy. Färkkilä and Laitinen report a case of heterotopic pregnancy where ectopic rupture at 19 weeks' gestation led to hypotension-induced brain damage in the intrauterine fetus, resulting in elective termination of this pregnancy [26].

In conclusion, whilst tachycardia is a useful clinical sign of haemoperitoneum when present, its absence should not reassure the clinician that large-volume haemoperitoneum is not present, particularly when normocardia is coupled with relatively low blood pressure. Implications for the diagnostic and therapeutic algorithm are significant; the urgency of the need for emergency surgical intervention may be no less in a normocardic patient than in a markedly tachycardic one.

## Figures and Tables

**Table 1:** Observations/vital signs.

Time	1105	1120	1150	1230	1230	1255	1320	1445	1450	1505	1720	1745
Location of patient	On scene	En route to hospital	Emergency department	Emergency department	Emergency department	Emergency department	Emergency department	Early pregnancy unit	Early pregnancy unit	Early pregnancy unit	Early pregnancy unit	Anaesthetic room

Respiratory rate (breaths per minute)	20	20	16	25	N/R	16	16	N/R	20	N/R	21	N/R

Oxygen saturation in air (%)	100	100	99	97	N/R	100	100	94	98	N/R	N/R	N/R

Heart rate (bpm)	70	70	112	69	N/R	71	69	71	62	73	82	86

Blood pressure (mmHg)	108/71	106/61	115/59	93/57 (right)	94/61 (left)	105/68	101/79	100/63	117/72	97/68	102/68	108/62

Colour	Normal	Normal	N/A	N/A	N/A	N/A	N/A	N/A	N/A	N/A	N/A	N/A

Conscious level	Alert	Alert	Alert	Alert	Alert	Alert	Alert	Alert	Alert	Alert	Alert	N/R

Blood sugar level (mmol/L)	5.7	5.7	N/A	N/A	N/A	N/A	N/A	N/A	N/A	N/A	N/A	N/A

Temperature (°C)	36.5	N/R	36.1	35.6	N/R	35.2	36.2	N/R	N/R	36.2	36.7	N/R

Pain score (0-10 scale)	10	8	N/A	N/A	N/A	N/A	N/A	N/A	N/A	N/A	N/A	N/A

National Early Warning System (NEWS) score	N/A	N/A	2	6	U	U	1	U	U	U	U	U

*Key*

N/A = not applicable (colour not routinely recorded on NEWS chart; blood sugar level not routinely recorded on NEWS chart; pain score not routinely recorded on NEWS chart; NEWS score only used in hospital).

N/R = not recorded.

U = Unable to calculate due incomplete documentation of observations.

## References

[1] (2012). *Advanced Trauma Life Support (ATLS) Student Course Manual*.

[2] Knight M., Nair M., Tuffnell D., Shakespeare J., Kenyon S., Kurinczuk J. J., on behalf of MBRRACE-UK (2017). *Saving Lives, Improving Mothers’ Care - Lessons learned to inform maternity care from the UK and Ireland Confidential Enquiries into Maternal Deaths and Morbidity 2013–15*.

[3] DeVoe R. W., Pratt J. H. (1948). Simultaneous intrauterine and extrauterine pregnancy. *American Journal of Obstetrics & Gynecology*.

[4] Maymon R., Shulman A. (1996). Controversies and problems in the current management of tubal pregnancy. *Human Reproduction Update*.

[5] Habana A., Dokras A., Giraldo J. L., Jones E. E. (2000). Cornual heterotopic pregnancy: Contemporary management options. *American Journal of Obstetrics & Gynecology*.

[6] Farquhar C. M. (2005). Ectopic pregnancy. *The Lancet*.

[7] Molloy D., Deambrosis W., Keeping D., Hynes J., Harrison K., Hennessey J. (1990). Multiple-sited (heterotopic) pregnancy after in vitro fertilization and gamete intrafallopian transfer. *Fertility and Sterility*.

[8] Practice Committee of American Society for Reproductive Medicine (2013). Medical treatment of ectopic pregnancy: a committee opinion. *Fertility and Sterility*.

[9] Sucov A., Deveau L., Feola P., Sculli L. (1995). Heterotopic pregnancy after in vitro fertilization. *The American Journal of Emergency Medicine*.

[10] Talbot K., Simpson R., Price N., Jackson S. R. (2011). Heterotopic pregnancy. *Journal of Obstetrics & Gynaecology*.

[11] Demetriades D., Chan L. S., Bhasin P. (1998). Relative bradycardia in patients with traumatic hypotension. *Journal of Trauma - Injury Infection and Critical Care*.

[12] Thompson D., Adams S. L., Barrett J. (1990). Relative bradycardia in patients with isolated penetrating abdominal trauma and isolated extremity trauma. *Annals of Emergency Medicine*.

[13] Adams S. L., Greene J. S. (1986). Absence of a tachycardic response to intraperitoneal hemorrhage. *The Journal of Emergency Medicine*.

[14] Jansen R. P. S. (1978). Relative bradycardia: a sign of acute intraperitoneal bleeding. *Australian and New Zealand Journal of Obstetrics and Gynaecology*.

[15] Snyder H. S. (1990). Lack of a tachycardic response to hypotension with ruptured ectopic pregnancy. *The American Journal of Emergency Medicine*.

[16] Öberg B., Thorén P. (1970). Increased activity in vagal cardiac afferents correlated to the appearance of reflex bradycardia during severe hemorrhage. *Acta Physiologica Scandinavica*.

[17] Mathlouthi N., Ghodbane I., Slimani O. (2012). Correlation between vital signs and hemoperitoneum in ruptured ectopic pregnancy. *La Tunisie Médicale*.

[18] Somers M. P., Spears M., Maynard A. S., Syverud S. A. (2004). Ruptured heterotopic pregnancy presenting with relative bradycardia in a woman not receiving reproductive assistance. *Annals of Emergency Medicine*.

[19] Cobb T. L. (1982). Orthostatic tachycardia and ectopic pregnancy: Normal pulse rate in the presence of massive hemorrhage. *Annals of Emergency Medicine*.

[20] Vayer J. S., Henderson J. V., Bellamy R. F., Galper A. R. (1988). Absence of a tachycardic response to shock in penetrating intraperitoneal injury. *Annals of Emergency Medicine*.

[21] Ley E. J., Salim A., Kohanzadeh S., Mirocha J., Margulies D. R. (2009). Relative bradycardia in hypotensive trauma patients: A reappraisal. *Journal of Trauma - Injury Infection and Critical Care*.

[22] Rana M. S., Khalid U., Law S. (2010). Paradoxical bradycardia in a patient with haemorrhagic shock secondary to blunt abdominal trauma. *BMJ Case Reports*.

[23] Hick J. L., Rodgerson J. D., Heegaard W. G., Sterner S. (2001). Vital signs fail to correlate with hemoperitoneum from ruptured ectopic pregnancy. *The American Journal of Emergency Medicine*.

[24] Rajoria L., Verma A., Hooja N., Hemani S., Verma K., Moolchandani R. (2013). Active management of third stage of labour in low resource setting: sublingual misoprostol or intramuscular oxytocin. *Indian Journal of Medical Case Reports*.

[25] Liumbruno G., Bennardello F., Lattanzio A., Piccoli P., Rossetti G. (2009). Recommendations for the transfusion of red blood cells. *Blood Transfusion*.

[26] Färkkilä A., Laitinen L. (2016). Heterotopic pregnancy during the second trimester is a severe complication of pregnancy. *Duodecim*.

